# Prevalence and Correlates of Active Amphetamine-Type Stimulant Use Among Female Sex Workers in Malaysia

**DOI:** 10.3389/fpsyt.2022.879479

**Published:** 2022-06-14

**Authors:** Courtney J. Pedersen, Jeffrey A. Wickersham, Frederick L. Altice, Adeeba Kamarulzaman, Kaveh Khoshnood, Britton A. Gibson, Antoine Khati, Francesca Maviglia, Roman Shrestha

**Affiliations:** ^1^Section of Infectious Diseases, Department of Internal Medicine, Yale School of Medicine, New Haven, CT, United States; ^2^Centre of Excellence for Research in AIDS (CERiA), Faculty of Medicine, University of Malaya, Kuala Lumpur, Malaysia; ^3^Department of Epidemiology of Microbial Diseases, Yale School of Public Health, New Haven, CT, United States; ^4^Department of Allied Health Sciences, University of Connecticut, Storrs, CT, United States

**Keywords:** amphetamine-type stimulant, substance use, sexual risks, HIV, sex worker, Malaysia

## Abstract

The use of amphetamine-type stimulants (ATS) has been associated with increased sexual risk behaviors and HIV transmission, among other adverse health outcomes. However, ATS use among female sex workers (FSWs) in Malaysia has not yet been characterized. We examined the prevalence and correlates associated with ATS use among Malaysian FSW. Between February and December 2016, 492 FSWs, including cisgender (*n* = 299) and transgender (*n* = 193) women, were recruited using respondent-driven sampling in Greater Kuala Lumpur, Malaysia. A structured questionnaire was used to collect demographic characteristics, sexual behaviors, ATS and other substance use, behavioral health issues, involvement in criminal justice, and experience of physical and sexual trauma. Logistic regression analyses were conducted to determine factors associated with active ATS use, defined as ATS use in the last 30 days. Nearly one-third (32.3%) of participants reported active ATS use. In the multivariable model, ATS use was associated with drug use during sex work (aOR = 17.10; 8.32–35.15), having moderate to severe level of substance use disorder (aOR = 3.38; 1.48–7.70), and engaging in sex work with multiple clients per day (two clients: aOR = 3.39; 1.36–8.46; three clients: aOR = 5.06; 1.81–14.10). A high prevalence of ATS use was documented in our sample. The presence of moderate to severe substance use disorder, the use of drugs during sex work activity, and having multiple sex work clients per day were significantly associated with active ATS use. Given these findings, prevention and harm reduction strategies need to be tailored to address the increasing ATS use and the associated adverse health consequences among FSWs in Malaysia.

## Introduction

Amphetamine-type stimulants (ATS) are synthetic psychostimulants such as methamphetamine, amphetamine, and ecstasy (MDMA), which can be injected, inhaled, smoked, or taken orally. Over the last decade, the global use of ATS has increased significantly (1). ATS are easy to produce, inexpensive to purchase, and hard to control, and are thus consumed in almost every region of the world, including South-East Asia. The growth in supply has led to decreases in prices of ATS throughout the region, increasing their affordability and popularity, with more drug users shifting from opioids to ATS (2). In Malaysia, recent statistics have shown an increase in ATS users, including its markets, seizures, consumption level, and manufacturing (1, 3). The rising consumption of ATS has implications for the prevention and harm reduction strategies targeting FSWs in Malaysia.

Global research on female sex workers (FSWs) indicates a shift to ATS use and away from injection drugs, primarily motivated by sex-work-related or occupation considerations. FSWs report using ATS for various reasons, including increased energy, enhanced libido, and better weight control (4–11). For example, FSWs reported that taking *yaba*, a pill form of methamphetamine and caffeine, allowed them increased stamina for working long hours, seeing more clients, charging higher prices, and being more sociable (4, 12, 13). Moreover, ATS may have a disinhibiting effect on sexual decision-making, so FSWs are more likely to engage in risky sexual behaviors (e.g., group sex, condomless sex, multiple sexual partners) after using ATS, all of which increase the risk of HIV and other STIs (5–7, 14–16).

The prevalence of HIV in FSWs is among the highest in all key populations in Malaysia. Recent data indicates that sex workers, including transgender and cisgender FSWs, are at heightened risk for HIV acquisition and transmission (17). A variety of behavioral, biological, and structural factors mediate this risk and contribute to an increased HIV prevalence in this population (18), with both shared and differing impacts between cisgender and transgender women. More recently, drug use, particularly ATS, has emerged as a potentially significant problem among FSWs in Malaysia (19) and, therefore, requires attention. However, limited exploration of ATS use related to HIV risks has been undertaken among FSWs specifically, where drug use and sex work are harshly criminalized. In Malaysia, both Sharia Law and the Civil Penal Code criminalize “solicitation for the purposes of prostitution” and both statutes are frequently applied to surveil, police, charge, and incarcerate sex workers (20), affecting their ability to safely access HIV prevention services (18). Therefore, the mixture of gender power dynamics, stigma, discrimination, and harsh criminalization of sex work and drug use complicates data collection and harm reduction efforts, which require a nuanced understanding of these populations and the specific risks they face (21–25).

As ATS use becomes more prevalent, it is crucial to characterize the impact of ATS use on marginalized groups, such as FSWs. This study examined the prevalence and factors associated with ATS use among FSWs in Malaysia. This paper is the first step toward developing a better understanding of ATS use among this HIV key population. We hope it will provide critical data required to develop future harm reduction efforts targeting this group.

## Methods

### Participants and Recruitment

Data were drawn from a cross-sectional study of 492 FSWs in Greater Kuala Lumpur, Malaysia, conducted between February and December 2016. Participants were recruited using respondent-driven sampling (RDS) from three distinct regions around Greater Kuala Lumpur, including communities of sex workers in urban, suburban, and coastal areas. A total of 28 “seed” participants who were FSWs working in various locations throughout Greater Kuala Lumpur and who had social connections in the sex working community were selected based on recommendations from community-based organizations serving sex workers, with attention given to gender and geographic representation.

After completing HIV and STI testing and questionnaires, each seed participant was given three coupons to recruit potential peers. Subsequent participants were, in turn, given three coupons to recruit additional peers. All participants provided informed consent prior to initiating study activities. Participants were eligible if they met all of the following criteria: a) were 18 years of age or older; b) identified as a cisgender woman (CW) or transgender woman (TW); c) had exchanged sex for money, goods, or services within the past 90 days; d) were willing to undergo HIV and STI testing; e) spoke either Malay, Tamil, or English; and f) were able to provide informed consent. Trained research assistants administered questionnaires that took ~60 min to complete. Participants received 50 Malaysian Ringgit (MYR) (~USD 17) for study participation and an additional 20 MYR (~ USD 7) for each of up to three peers they successfully recruited into the study.

### Measures

#### Dependent Variable

The primary outcome variable, active ATS use, was defined as any use of amphetamine, methamphetamine, or MDMA (3,4-methylenedioxymethamphetamine), commonly known as “ecstasy,” in the last 30 days.

#### Demographic Characteristics

Measures of socio-demographics included age, ethnicity, gender identity, monthly income, and housing status.

#### Sex Work Characteristics

Total time involved in sex work was measured as the total number of years having worked in sex work. Hours per week engaged in sex work was defined as the weekly number of hours spent soliciting sex work clients and involved in sex work. The number of sex work clients per day (during the last 30 days) was measured as the number of individuals the participant engaged in sex work within a 24-h period. Any condomless receptive vaginal or anal sex with a client in the last 30 days and sex work debut prior to the age of 18 years were also measured. Lastly, we assessed the co-occurrence of drug use during sex work by asking FSWs about using any illicit drugs immediately prior to or during sexual activity with a client in the last 30 days.

#### Lifetime and Active Alcohol and Drug Use

Lifetime and active alcohol and drug use were assessed. Lifetime drug use was defined as having ever used a substance one or more times in the entire course of life, and “active” drug use was defined as having used a substance in the 30 days prior to the interview. The most commonly used route of administration for each drug used was also measured, including smoking, oral (eating or swallowing), injecting, or sniffing.

#### Behavioral Health Measures

The Drug Abuse Screening Test (DAST-10) (26) was used to measure the degree of drug-related problems experienced by participants. Standard cutoff scores to stratify the degree of drug-related problems were used, including: low (1, 2), moderate (3–5), substantial (6–8), and severe (9, 10). The Center for Epidemiologic Studies-Depression (CES-D) was used to screen for depression symptoms, using the standard cutoff score of ≥10 to indicate the presence of moderate to severe depression (27, 28). History of suicidality was measured with a single-item question, “*Have you ever thought about killing yourself or tried to kill yourself* ,” with a binary response (“*yes*” or “*no*”).

#### Physical and Sexual Trauma

Four items from the U.S. Centers for Diseases Control and Prevention's Behavioral Risk Factor Surveillance System questionnaire (29) were used to measure the experience of childhood and adulthood physical and sexual trauma. Childhood physical trauma was measured with a single-item question, “Before the age of 18, were you ever hit, slapped, kicked, or physically hurt by an adult?”). Childhood sexual trauma was measured with two items: “*Before the age of 18, were you ever forced to have sex by an adult or older child?*” and “*Before the age of 18, were you ever touched in a sexual way by an adult or older child when you did not want to be touched that way or were you ever forced to touch an adult or older child in a sexual way?*” A “*yes*” response to either question resulted in a “*yes*” coding for the presence of childhood sexual trauma. Adulthood physical trauma was measured with a single-item question, “*Since the age of 18, have you ever been hit, slapped, kicked, or physically hurt by an adult?*” Adulthood sexual trauma was measured with the single-item question, “*Since the age of 18, have you ever had any unwanted sexual experiences?*” Each variable was coded “*yes*” or “*no*”.

#### Criminal Justice History

Two levels of previous involvement in the criminal justice system were measured. “Previously jailed” was defined as any prior placement in detention, jail, or lock-up by law enforcement. “Previously incarcerated” was defined as any prior imprisonment or incarceration following conviction of a criminal offense.

#### Analytic Approach

Descriptive statistics were calculated, including frequencies and percentages for categorical variables and means and standard deviations for continuous variables. Bivariate analyses were performed using Chi-square/Fisher's exact and Student's *t*-tests for categorical and continuous covariates, respectively, to identify factors associated with the outcomes (ATS use). A multivariable logistic regression model included variables with bivariate associations of *p* < 0.05. Estimates were evaluated for statistical significance based on 95% confidence intervals and statistical significance of *p* < 0.05. Test for multicollinearity was performed and no issues were detected. All statistical analyses were performed using SPSS version 26.

## Results

### Participant Characteristics

Participant characteristics are presented in Table 1. A total of 492 FSWs–CW (60.8%) and TW (39.2%)–were enrolled from three sites in the Greater Kuala Lumpur area: Metropolitan Kuala Lumpur (54.9%); Klang (31.9%); and Petaling Jaya (13.2%). Over half of the participants were in the age group 26–40 years (50.4%). The majority were stably housed in a house or apartment with roommates (89.0%), about one-third (36.2%) had reached the secondary education level or higher, and 26.0% reported making < RM 1,000 per month (~USD 244).

**Table 1:** Bivariate and multivariable logistic regression for active ATS use (*N* = 492).

	**%(*n*) or M(SD)**	**OR (95%CI)**	***P***	**aOR (95% CI)**	***p***
**Sociodemographic**					
Interview site					
Metropolitan Kuala Lumpur	54.9 (270)	5.01 (3.23, 7.78)	<0.001	1.21 (0.43, 3.42)	0.717
Klang	31.9 (157)	0.65 (0.36, 1.18)	0.156		
Petaling Jaya	65 (13.2)	0.17 (0.10, 0.29)	<0.001	0.54 (0.17, 1.73)	0.297
Ethnicity					
Malay	54.9 (270)	2.33 (1.57, 3.47)	<0.001	1.08 (0.55, 2.17)	0.828
Indian	22.2 (109)	0.79 (0.50, 1.26)			
Gender identity					
Cisgender	60.8 (299)	0.94 (0.64, 1.38)	0.748		
Transgender	39.2 (193)	1.07 (0.72, 1.57)	0.748		
Age (years)					
18–25	13.4 (66)	1.42 (0.77, 2.63)	0.258		
26–40	50.4 (248)	1.47 (0.78, 2.76)	0.237		
41+	36.2 (178)	Reference	−		
Monthly income <1,000 MYR	26.0 (128)	0.81 (0.52, 1.52)	0.338		
Unstable housing	11.0 (54)	3.56 (1.99, 6.37)	<0.001	1.59 (0.64, 3.97)	0.322
**Sex work**					
Total time in sex work (in years)	12.1 (10.4)	1.02 (1.01, 1.04)	0.037	1.05 (0.95, 1.15)	0.378
Sex work debut pre-18 years of age	16.1 (79)	1.44 (0.87, 2.37)	0.152		
Hours worked per day (last 30 days)	6.0 (3.3)	1.09 (1.03, 1.15)	0.004	1.03 (0.95, 1.12)	0.475
Number of clients per day					
One	22.8 (112)	Reference	−		
Two	36.6 (180)	3.25 (1.81, 5.84)	<0.001	3.39 (1.36, 8.46)	0.009
Three	18.5 (91)	4.28 (2.23, 8.22)	<0.001	5.06 (1.81, 14.10)	0.001
Four or more	22.2 (109)	2.08 (1.08, 3.99)	0.029	2.65 (0.91, 7.78)	0.075
Condomless receptive sex with client	68.3 (336)	1.07 (0.71, 1.60)	0.743		
Drug use during sex work (last 30 days)	33.5 (165)	48.12 (27.59, 83.92)	<0.001	17.10 (8.32, 35.15)	<0.001
**Criminal justice history**					
Previously jailed (lock-up)	49.2 (242)	8.85 (5.57, 14.04)	<0.001	1.50 (0.66, 3.40)	0.332
Previously incarcerated (prison)	32.7 (161)	8.72 (5.67, 13.43)	<0.001	1.23 (0.54, 2.78)	0.622
**Behavioral health**					
DAST-10					
None	44.3 (218)	Reference	−	Reference	−
Low level	20.5 (101)	1.98 (1.07,3.65)	0.029	1.04 (0.43, 2.54)	0.935
Moderate to severe level	27.4 (135)	30.13 (17.07,53.19)	<0.001	3.38 (1.48, 7.70)	0.004
Depression symptomatic	57.1 (281)	1.48 (1.00,2.18)	0.048	0.76 (0.37, 1.54)	0.439
History of suicidality	20.9 (388)	1.68 (1.07, 2.63)	0.023	0.92 (0.43, 1.94)	0.822
**Physical and sexual trauma**					
Childhood physical trauma	34.8 (171)	2.15 (1.46, 3.17)	<0.001	1.46 (0.38, 1.58)	0.283
Childhood sexual trauma	36.8 (181)	1.67 (1.13, 2.47)	0.010	0.78 (0.73, 2.93)	0.484
Adulthood physical trauma	33.7 (166)	2.05 (1.38, 3.03)	<0.001	1.07 (0.53, 2.19)	0.847
Adulthood sexual trauma	17.3 (85)	1.70 (1.06, 2.75)	0.029	0.97 (0.43,2.22)	0.944

Regarding sex work characteristics, participants had engaged in sex work for an average of 12.1 (SD = 10.4) years, with 16.1% reporting first involvement in sex work before the age of 18. During the last 30 days, participants worked for an average of 6 h per day (SD = 3.3), with the majority of them engaging in sex with two or more clients per day (77.2%). Over two-thirds of participants (68.3%) reported condomless receptive sex with clients, and about one-third (33.5%) reported drug use during sex work in the last 30 days.

**Figure 1:**
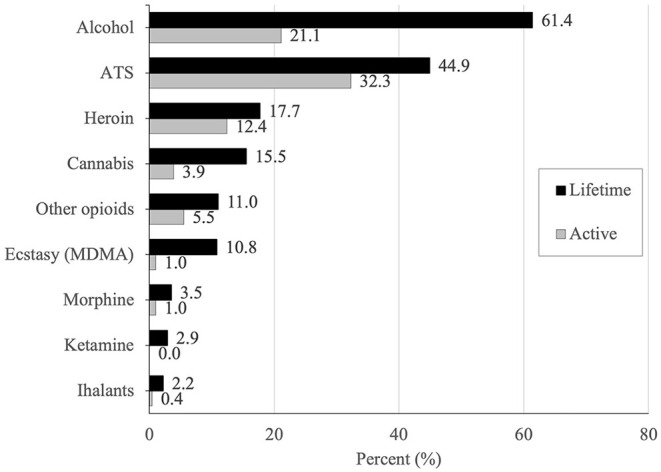
Lifetime and active history of substance use among sex workers who are cisgender or transgender women, in Greater Kuala Lumpur, Malaysia.

The history of substance use is shown in Figure 1. The majority (72.2%) of the participants reported using any substance ever in their lifetime. The most commonly reported substance used across the lifetime was alcohol (61.4%), followed by ATS (44.9%), heroin (17.7%), and cannabis (15.5%). Nearly half (49.2%) of participants had used a substance in the last 30 days. The most commonly used drug in the last 30 days was ATS (32.3%), followed by alcohol (21.2%), heroin (12.4%), other opioids (5.5%), and cannabis (3.9%). The nearly universal route of administration for ATS was smoking (98.2%), with only 1 participant reporting prior injection of ATS (0.5%). Roughly one-third (27.4%) of participants screened positive for moderate to severe drug abuse disorders. Over half of participants (57.1%) met screening criteria for having moderate to severe depression, and 20.9% had a history of active suicide ideation or suicide attempts.

Most participants (57.5%) reported having been involved with the criminal justice system (CJS) in their lifetime, including prison (32.7%) and detention centers (49.2%). The most common reason for imprisonment was sex work (66.8%) and drug use (40.3%). Regarding physical and sexual trauma, over one-third reported having experienced physical assault (34.8%) and sexual assault (36.8%) in their childhood, whereas 33.7% and 17.3% reported physical and sexual assault in their adulthood, respectively.

### Correlates of Active ATS Use

Table 1 shows the independent correlates associated with active ATS use in the multivariate logistic regression. Use of drugs during sex work in the last 30 days (aOR = 17.10; 95% CI, 8.32–35.15), having moderate to severe level of substance use disorder (aOR = 3.38; 95% CI, 1.48–7.70), and engaging in sex work with multiple clients per day (two clients: aOR = 3.39; 95% CI, 1.36–8.46; three clients: aOR = 5.06; 95% CI, 1.81–14.10) were all significantly associated with active ATS use in the multivariable model.

## Discussion

The use of ATS among FSWs in Malaysia, and associated factors, have been scantly documented in Malaysia. To the best of our knowledge, this is the first study to assess ATS use and the associated factors among Malaysian FSW, a setting where FSWs are highly stigmatized and discriminated against (20), and consequently face challenges accessing healthcare and HIV prevention (30). This study provides important insights into the patterns of drug use, particularly ATS, among this highly marginalized group. Specifically, our findings indicate a high prevalence of active ATS use in this sample. Although the disaggregated ATS use data is not available, estimates have shown an increase in ATS users based on seizures, consumption level, and manufacturing in the recent years (1, 31), which has important implications for the prevention and harm reduction strategies targeting FSWs in the region. These results are similar to those observed among FSWs elsewhere in Asia and globally (8, 19, 32, 33). This finding is particularly concerning in the Malaysian context because Malaysia serves as a transit country for drug trafficking, and increased ATS pill seizures by law enforcement in recent years suggest the increasing availability of ATS in the region (1). With a large proportion of Malaysian FSWs using ATS, prevention strategies and health policies need to be tailored to these contexts to address the increasing use of ATS and the associated adverse health consequences.

Participants in our sample also faced several vulnerabilities to HIV, including condomless sex, entering sex work before 18 years of age, and having multiple sex work clients, contributing to the ongoing HIV epidemic among sex workers. Worsening the concern of sexual risk is the issue of drug use, particularly during sex work. Our findings revealed that a high proportion (72.2%) of FSWs in the study reported ongoing substance use. Over one-third of participants reported substance use during sex work, demonstrating that drug use was largely part of the occupational culture of sex work. Additionally, the use of ATS was significantly associated with having multiple sex clients per day, a finding that is consistent with that from prior studies (5–7, 14, 15). Although not explored here, prior literature has shown that ATS is not only used for recreational and social purposes to increase libido, reduce sexual inhibitions, enhance sexual pleasure, enhance sociability, but also for occupational purposes in order to increase energy and productivity (4–9, 11–13, 18, 34), thus facilitating longer work hours, and enabling women to see more clients and potentially increase their income.

Furthermore, the literature has shown that ATS use is associated with impaired decision making, prolonged sexual activities, lack of negotiation for safer sex, and inconsistent condom use among many groups, including FSWs (4–6, 16, 35–37). As drug use can increase risk-taking behaviors, occupationally driven drug use should be addressed as part of HIV prevention efforts. The high levels of ATS use and its influence on HIV transmission indicate the need to address it as an important risk factor in this group. Prevention efforts, therefore, need to be focused on sexual behaviors and increasing understanding of the risk associated with drug use, especially ATS.

Interestingly, however, while receptive condomless sex with a client was frequent and concerned two-thirds of our sample, it was not associated with ATS use, suggesting that condomless sex in this community is driven by other factors. In contexts where sex work is criminalized, condom use is impeded by a variety of barriers including dangers of disclosure of sex worker status to a health professional; lack of negotiating power with clients; and legal liabilities connected to possession of condoms (18). In a qualitative case study assessing the impact of sex work criminalization laws, for instance, Malaysian sex workers reported that carrying condoms is used by police as evidence of engagement in sex work to arrest presumed sex workers (20). Modeling studies estimate that the decriminalization of sex work could lead to a global 33–46% reduction in new HIV infections in sex workers over 10 years (18). Absent a change in legal status, however, community-driven interventions such as peer outreach and education have been shown to be successful in increasing access to safe sex supplies and self-efficacy in negotiating condom use (34, 38, 39).

There are several potential limitations to this study. While the RDS sampling was beneficial in obtaining a large sample size from this hard-to-reach population, it is possible that bias was introduced due to social networks. We attempted to prevent this as much as possible by sampling from three sites with unique geographic characteristics within the Greater Kuala Lumpur area and including multiple seed participants at the beginning of the study. Additionally, the topics of our study focused on both culturally sensitive and criminalized behaviors; as such, our results may have been influenced by social desirability reporting bias. Due to the scope of this study, we focused on correlates of ATS use in this study. Future research should explore whether the engagement in HIV risk behavior among ATS users differs by gender identity or other demographic characteristics. Lastly, given the cross-sectional design of our study, we could not determine the temporality or causality of any of the variables that were independently associated with active ATS use.

Despite these limitations, recognition, and understanding of ATS use and HIV-related risks surrounding this group could lead to prevention efforts that will ultimately help slow down the transmission of HIV within this vulnerable population. Policing, supply reduction, and zero tolerance policies currently drive the response to ATS use in the Southeast Asia region. These approaches, however, have been shown to be at best ineffective and at worst harmful by driving incarceration and increasing social vulnerability (40). There is an urgent need for harm reduction services, which have typically been focused on opioid users, to be tailored to the needs of ATS users. For example, education to increase awareness on some of the risks associated with ATS use and measures to alleviate them, strengthened psychosocial support, distribution of safe smoking and safe sex kits, and substitution treatment for amphetamine dependence using dexamphetamine, modafinil or milder plant-bases stimulants are examples of harm reduction-informed strategies to engage sex workers who use ATS (40–42).

## Conclusion

The current study constitutes one of the first efforts toward determining the prevalence and factors associated with ATS use among Malaysian FSWs, a group that already faces substantial occupational risk and societal marginalization. The results highlight a high prevalence of substance use, particularly ATS, among Malaysian FSWs. The findings further offer important insights into the intersection of ATS use, substance use-related problems, and HIV risk for prevention efforts tailored for FSWs, and sex workers in general, in Malaysia and elsewhere. There is an urgent need for tailored multilevel interventions to mitigate the impact of the dual epidemic of substance use and HIV on this highly marginalized community.

## Data Availability Statement

The raw data supporting the conclusions of this article will be made available by the authors, without undue reservation.

## Ethics Statement

The Institutional Review Board (IRB) at Yale University and the Ethics Committee at the University of Malaya approved the study. The participants provided their written informed consent to participate in the study.

## Author Contributions

CP, JW, and RS: study conception and design, analysis, and interpretation of results. JW: data collection. CP, JW, RS, FM, and AKh: draft manuscript preparation. All authors reviewed the results and approved the final version of the manuscript.

## Funding

This work was supported by the National Institute on Drug Abuse for career development under Grant [K24 DA017072], [K01 DA051346], and [K01 DA038529].

## Conflict of Interest

The authors declare that the research was conducted in the absence of any commercial or financial relationships that could be construed as a potential conflict of interest.

## Publisher's Note

All claims expressed in this article are solely those of the authors and do not necessarily represent those of their affiliated organizations, or those of the publisher, the editors and the reviewers. Any product that may be evaluated in this article, or claim that may be made by its manufacturer, is not guaranteed or endorsed by the publisher.
